# From genome to toxicity: a combinatory approach highlights the complexity of enterotoxin production in *Bacillus cereus*

**DOI:** 10.3389/fmicb.2015.00560

**Published:** 2015-06-10

**Authors:** Nadja Jeßberger, Viktoria M. Krey, Corinna Rademacher, Maria-Elisabeth Böhm, Ann-Katrin Mohr, Monika Ehling-Schulz, Siegfried Scherer, Erwin Märtlbauer

**Affiliations:** ^1^Department of Veterinary Sciences, Faculty of Veterinary Medicine, Ludwig-Maximilians-Universität MünchenOberschleißheim, Germany; ^2^Lehrstuhl für Mikrobielle Ökologie, Zentralinstitut für Ernährungs- und Lebensmittelforschung, Wissenschaftszentrum Weihenstephan, Technische Universität MünchenFreising, Germany; ^3^Functional Microbiology, Department of Pathobiology, Institute of Microbiology, University of Veterinary Medicine ViennaVienna, Austria

**Keywords:** *Bacillus cereus*, enterotoxins, Nhe, Hbl, host cell cytotoxicity, food poisoning

## Abstract

In recent years *Bacillus cereus* has gained increasing importance as a food poisoning pathogen. It is the eponymous member of the *B. cereus sensu lato* group that consists of eight closely related species showing impressive diversity of their pathogenicity. The high variability of cytotoxicity and the complex regulatory network of enterotoxin expression have complicated efforts to predict the toxic potential of new *B. cereus* isolates. In this study, comprehensive analyses of enterotoxin gene sequences, transcription, toxin secretion and cytotoxicity were performed. For the first time, these parameters were compared in a whole set of *B. cereus* strains representing isolates of different origin (food or food poisoning outbreaks) and of different toxic potential (enteropathogenic and apathogenic) to elucidate potential starting points of strain-specific differential toxicity. While toxin gene sequences were highly conserved and did not allow for differentiation between high and low toxicity strains, comparison of *nheB* and *hblD* enterotoxin gene transcription and Nhe and Hbl protein titers revealed not only strain-specific differences but also incongruence between toxin gene transcripts and toxin protein levels. With one exception all strains showed comparable capability of protein secretion and so far, no secretion patterns specific for high and low toxicity strains were identified. These results indicate that enterotoxin expression is more complex than expected, possibly involving the orchestrated interplay of different transcriptional regulator proteins, as well as posttranscriptional and posttranslational regulatory mechanisms plus additional influences of environmental conditions.

## Introduction

*Bacillus cereus* has become a hygienic and technological problem of increasing importance in the food industry. It is ubiquitous, produces heat resistant endospores and is able to form biofilms (Wijman et al., 2007; Stenfors Arnesen et al., 2008; Nam et al., 2014). Due to its lipo- and proteolytic properties it plays an important role in food spoilage (Andersson et al., 1995), but the main problem is the production of toxins, which are responsible for food poisoning. In 2011, the number of *B. cereus*-associated food poisoning outbreaks within the European Union increased by 122% (Anonymous, 2013). Various efforts have been made to predict the toxic potential of newly isolated strains. The *B. cereus sensu lato* group consists of eight closely related species, i.e., *B. cereus, Bacillus anthracis, Bacillus thuringiensis, Bacillus weihenstephanensis, Bacillus mycoides, Bacillus pseudomycoides, Bacillus cytotoxicus*, and *Bacillus toyonensis*. Highly toxic as well as non-toxic strains exist among most of these species. Guinebretière et al. (2008) further divided the *B. cereus* group into seven phylogenetic groups and subgroups (Guinebretière et al., 2008). For *B. cereus sensu lato*, the most commonly used method in routine diagnostics is the detection and quantification of colonies on selective culture media such as PEMBA or MYP according to international standards (Ehling-Schulz and Messelhäusser, 2013), which does not allow to distinguish the species of the *B. cereus* group. Therefore, only *presumptive B. cereus* can be detected (ISO 7932). While molecular methods for quantification of *B. cereus* have been established, no differentiation between living and dead cell or between spores and vegetative cells could be achieved (Martinez-Blanch et al., 2009; Ceuppens et al., 2010; Dzieciol et al., 2013). Currently, the molecular detection of toxin genes rather than species differentiation is applied (Ehling-Schulz and Messelhäusser, 2013). Toward this end, multiplex PCR systems for the detection of *nhe, hbl, cytK*, and *ces* have been established (Guinebretière et al., 2002; Fricker et al., 2007; Wehrle et al., 2009). However, the presence or absence of toxin genes does not allow to reliably infer the toxic potential, as highly variable amounts of toxins are produced in strains sharing the same toxin genes (Dietrich et al., 2005; Jeßberger et al., 2014).

A peptide synthetase, encoded by *ces*, produces the emetic toxin cereulide, the causative agent of the emetic syndrome leading to vomiting (Ehling-Schulz et al., 2005b; Stenfors Arnesen et al., 2008). Due to its resistance against heat, acids and proteolysis, it can't be inactivated by heat treatment of contaminated food samples or during stomach passage (Ehling-Schulz et al., 2004). The amount of preformed cereulide present in foods can consequently be used as an indicator for the toxic potential and thus for the consumer's risk (Ehling-Schulz and Messelhäusser, 2013). Currently, an ISO method for quantitative detection of cereulide in food samples (EU-CEN action: TC 257/WG6) is under development.

Enteropathogenic *B. cereus* cause diarrhea due to the production of enterotoxins in the human intestine. This occurs after viable bacteria or most likely spores are ingested together with contaminated foods (Clavel et al., 2004; Ceuppens et al., 2012). So far, the two three component enterotoxin complexes Nhe (non haemolytic enterotoxin, Lund and Granum, 1996) and Hbl (haemolysin BL, Beecher et al., 1995) have been described, as well as the single protein CytK (cytotoxin K, Lund et al., 2000). Only very few strains bear the highly toxic variant CytK1 and these are classified as a separate species, *B. cytotoxicus* (Guinebretière et al., 2013). The *nhe* genes are present in all enteropathogenic *B. cereus* strains analyzed so far. The *hbl* operon is present in approximately 50% of the strains, whereas its prevalence seems to be higher in clinical and food isolates (Guinebretière et al., 2002; Ehling-Schulz et al., 2005a; Moravek et al., 2006). Prediction of toxicity is based on the quantification of the enterotoxin components in *B. cereus* culture supernatants. Currently, three test systems are commercially available, detecting the enterotoxin components Hbl L2, NheA, as well as NheB and Hbl L2, respectively. However, results may often be inappropriate for evaluating the risk of contaminated food samples, as the enterotoxins, unlike the emetic toxin cereulide, are largely produced in the intestine.

According to recent studies, further virulence factors such as sphingomyelinase, haemolysin II or exoproteases contribute to pathogenicity. A role of sphingomyelinase as a virulence factor against insects and murine intestinal epithelial cells as well as its interaction with Nhe have been reported (Doll et al., 2013). HlyII was shown to induce *in vitro* and *in vivo* apoptosis to macrophages (Tran et al., 2011). In another study, *hlyII* was preferably found in pathogenic *B. cereus*, and *inhA1* and *nprA* expression (both genes encoding metalloproteases) was higher in pathogenic than in non-pathogenic strains (Cadot et al., 2010).

So far, it is still not clear why toxin production and thus toxicity is so highly variable among different *B. cereus* strains. Thus, we aimed to explore whether distinct bacterial (genetic) factors, mechanisms or regulatory levels can be identified upon which high and low toxicity of enteropathogenic *B. cereus* strains can be distinguished unequivocally. Therefore, we chose a representative set of *B. cereus* isolates and characterized it with respect to enterotoxin gene presence, sequence and transcription as well as protein secretion, enterotoxin production and cytotoxicity.

## Materials and methods

### Bacterial strains, growth conditions, and sample preparation

All *B. cereus* strains used in this study are listed in Table 1 and Supplemental Table S1. Strains were grown at 30°C on CGY plates or in CGY broth. Unless stated otherwise, 30 ml cultures were inoculated from 17 h pre-cultures, and were grown in 300 ml Erlenmeyer flasks with 125 rpm at 30°C. For determination of enterotoxin production and toxicity, all strains listed in Table S1 were routinely grown for 6 h in CGY medium, inoculated with an optical density at 600 nm (OD_600_) of 0.2. For growth tests in this study, CGY medium was inoculated with a 17 h pre-culture to an OD_600_ of 0.05 and OD_600_ was recorded every 30 min. 5 milliliter (at 2 h) and 3 ml (at 6 h) samples were taken and centrifuged for 15 min at RT and 3500 rpm. Cell pellets were immediately frozen at −80°C and used for preparation of RNA and measurement of intracellular protein concentrations. Supernatants were filtered through 0.2 μm filters and separated for measurement of extracellular protein concentrations and enterotoxin production. For the latter, 1 mM EDTA was added to the culture supernatant. Supernatants were frozen and stored at −20°C. All growth tests and sample preparations were carried out in triplicates.

**Table 1:** **Set of the 19 *B. cereus* strains used in this study**.

***B. cereus* strain**	**Origin**		**Genotype clade (group)**	**Toxin gene profiling**								**Toxin titer NheB**	**Toxicity Vero**
				***ces***	***hbl***	***nhe***	***cytK2***	***hlyII***	***inhA1***	***nprA***	**profile**		
14294-3 (M6)	Ice cream	Food	I (III)	−	+	+	+	−	+	+	A	m	m
SDA KA96	Raw milk	Food	I (III)	−	+	+	+	−	+	+	A	hi	hi
INRA A3	Starch	Food	II (IV)	−	+	+	+	−	+	+	A	lo	lo
INRA C3	Past. Carrot	Food	II (IV)	−	+	+	+	−	+	+	A	hi	hi
6/27/S	Human feces	Diarrheal	II (IV)	−	+	+	+	−	+	+	A	m	m
F3175/03 (D7)	Human feces	Diarrheal	II (IV)	−	+	+	+	−	+	+	A	hi	m
RIVM BC 934	Lettuce	Food	II (IV)	−	+	+	+	−	+	+	A	lo	lo
F528/94	Beef and chow mein and rice, food poisoning outbreak	Diarrheal	I (II)	−	+	+	−	+	+	+	C	lo	lo
F837/76	Human, postoperative infection	*hbl* reference	I (III)	−	+	+	−	+	+	+	C	hi	hi
RIVM BC 126	Human feces	Diarrheal	I (II)	−	+	+	−	−	+	+	C	hi	hi
MHI86	Infant food	Food	I (III)	−	−	+	+	−	+	+	D	lo	lo
F4429/71	Vanilla pudding	Diarrheal	I (III)	−	−	+	+	−	+	+	D	hi	hi
RIVM BC 964	Kebab	Food	II (IV)	−	−	+	+	+	+	+	D	hi	hi
F3162/03 (D8) ^*^	Human feces	Clinical	I (III)	−	−	+	+	−	+	+	D	lo	hi
MHI226 ^^**^^	Milk and milk products	Food	I (III)	−	−	+	−	+	+	+	F	lo	lo
NVH 0075−95	Stew with vegetables, foodpoisoning	*nhe* reference	I (III)	−	−	+	−	−	+	+	F	hi	hi
WSBC10035	Past. Milk	Food	I (III)	−	−	+	−	−	+	+	F	hi	hi
RIVM BC 90	Human feces	Diarrheal	I (III)	−	−	+	−	−	+	+	F	lo	lo
7/27/S	Human feces	Diarrheal	I (III)	−	−	+	−	−	+	+	F	hi	hi

*Genotyping was performed by sequence analyses of the genetic markers spoIIIAB and panC, toxin profiling by PCR analyses using specific primers for ces, enterotoxin genes and virulence factors, toxin titers were determined by sandwich EIAs against NheB and toxicity was analyzed by WST-1-bioassay on Vero cells. Isolates were classified highly (hi), medium (m) or low (lo) toxic according to their cytotoxicity (hi: >500; m: 250-500; lo: <250) and NheB titers (hi: >4000; m: 2000-4000; lo: <2000)*.

* *, strain showed high toxicity but particularly low NheB titers due to binding failure of mab 2B11 in sandwich EIA (A. Didier, in preparation)*.

** *, sequence analysis revealed a truncated hbl operon; as strain is not able to produce Hbl L2 and Hbl B protein (negative in EIAs), it was allocated to profile F*.

### Cell lines and culture conditions

CaCo-2 cells were from DSMZ (German Collection of Microorganisms and Cell Cultures, Braunschweig, Germany) and Vero cells from ECACC (European Collection of Cell Cultures). Both cell lines were cultured in media recommended by the supplier and as described elsewhere (Jeßberger et al., 2014).

### Toxin gene profiling and sequence typing

Total DNA was isolated using the MasterPure™ Gram Positive DNA Purification Kit (Epicentre). Detection of *nhe, hbl, cytK* and *ces* was performed by multiplex PCR (Ehling-Schulz et al., 2006). The sporulation stage IIIAB gene (*spoIIIAB*) was used as a genetic locus for sequence typing and classification of *B. cereus* isolates to clades (Ehling-Schulz et al., 2005a; Ehling-Schulz and Messelhäusser, 2013). Therefore, sequences were aligned using CLUSTAL X software (Thompson et al., 2002) and cluster analysis of aligned sequences was performed by TREECON using UPGMA method for inferring tree topology (Van De Peer and De Wachter, 1997). Alternatively sequence typing and clade classification was carried out by using the pantothenate synthetase gene *panC* (Guinebretière et al., 2008).

### Comparison of toxin operon nucleotide and amino acid sequences

Nucleotide sequences of *nheABC* and *hblCDA* were obtained from a *de novo* genome sequencing approach (MiSeq® Reagent Kit v2, 500 cycles, Illumina) (Böhm et al., in preparation), identified via BLASTN algorithm and compared using CloneManager 7. Multiple amino acid sequence alignments of single Nhe and Hbl enterotoxin components were constructed with Clustal Omega v1.2.0 (Sievers et al., 2011). Signal peptide sequences were predicted using the SignalP 4.1 server (Petersen et al., 2011).

### RNA isolation, cDNA synthesis, and quantitative real-time PCR (qRT-PCR)

Isolation of total RNA and DNAse I digestion was performed as previously described (Dommel et al., 2010). Using the random primers of the qScript cDNA Supermix (Quanta Biosciences), first strand synthesis of 1 mg of total RNA was performed. qRT-PCR was done as reported repeatedly and the 2^−ΔΔ*C*_T_^ method was used for calculation of relative gene expression (Livak and Schmittgen, 2001; Lücking et al., 2009; Dommel et al., 2010). Primer amplification efficiencies (E) were in the desirable range (E = 1.7 − 2.1) for all primer pairs listed in Table S2 as evaluated according to the equation E = 10^(−1/slope)^ (Pfaffl et al., 2002). 16S *rrn* transcription levels determined by qRT-PCR (Martineau et al., 1996) served as the reference control for normalization applying the 2^−ΔΔ*C*_T_^ method (Livak and Schmittgen, 2001). This method is based on the following formula: amount of target transcript = 2^−ΔΔ*C*_T_^ with −ΔΔ*C*_T_ = − (Δ*C*_T(sample)_ − Δ*C*_T(calibrator)_) = −((*C*_T(reference gene)_ − *C*_T(target gene)_)_sample_ − (*C*_T(reference gene)_ − *C*_T(target gene)_)_calibrator_). *C*_T_ denotes the cycle number of the amplification reaction that exceeds the quantification threshold of the instrument. To determine the relative transcription of a target gene, *rrn*-normalized transcription level of a sample (Δ*C*_T(sample)_) was set relative to the transcript level of an external calibrator (Δ*C*_T(calibrator)_) and multiplied with 100 to obtain relative transcription in percent [%]. Expression level of the *hblD* gene of the toxin reference strain *B. cereus* F837/76 at 6 h served as calibrator that was set to 100% (log-2 = 0), all other transcript levels were compared to this condition using the 2^−ΔΔ*C*_T_^ method.

### Enzyme immunoassays (EIAs)

The enterotoxin components NheB and Hbl L2, L1 and B were detected in indirect and sandwich enzyme immunoassays as described elsewhere (Dietrich et al., 1999, 2005; Moravek et al., 2006). For NheB detection, the sandwich EIA using mabs 2B11 and 1E11-HRP or the indirect EIA using only mab 1E11 were performed. Titers were defined as the reciprocal of the highest dilutions resulting in an absorbance value of ≥1.0.

### WST-1 bioassays

WST-1 bioassays were performed as previously described (Dietrich et al., 1999, 2005; Didier et al., 2012; Jeßberger et al., 2014). Characterization of the toxic activity of the 136 *B. cereus* strains was carried out on Vero cells, as this assay is used in our routine *B. cereus* diagnostic. For the 19 strains in this study, the human colon carcinoma cell line CaCo-2 was further used. For detection, the optical density at 450 nm was measured with a Tecan photometer. With Ridawin software dose-response curves and thus 50% inhibitory concentrations were calculated, shown as reciprocal titers.

### Propidium iodide (PI) influx studies

Enterotoxin-induced pore formation in the membranes of CaCo-2 cells was measured by propidium iodide influx tests as described before (Jeßberger et al., 2014). For each *B. cereus* strain, a specific curve (increase of fluorescence per time) was obtained, of which the value of the highest linear slope was calculated. Strains were compared according to those values.

### Quantification of total protein amount

Extracellular proteins were obtained from culture supernatants. For isolation of intracellular proteins cell pellets were resuspended in 50 mM Tris, pH 7,5 and disrupted by two passages through a French Pressure cell press (2 kbar). The soluble proteins were collected by centrifugation (15.000 rpm, 45 min, 4°C). Intra- and extracellular protein concentrations were quantified by Roti-Nanoquant Kit (Roth) in microtiterplates according to the manufacturer's instructions. Colorimetric reactions were measured with Infinite F200 reader (Tecan) at wavelengths of 610/450 nm. Protein concentrations were determined by quotient of optical densities OD_610_/_450_ referred to internal standard generated by Quick Start Bovine Serum Albumin Standard (Biorad).

### Isolation of extracellular protein extracts for gelelectrophoretic separation

Protein extracts were precipitated by addition of ice-cold trichloroacetic acid solution to a final concentration of 10% (v/v) overnight at 4°C. Precipitated proteins were centrifuged (1 h, 10.000 rpm, 4°C) and protein pellet was washed 3 times with acetone. Pellets were dried overnight at 4°C and finally resuspended in DIGE lysis buffer (7 M Urea, 2 M Thiourea, 4% (w/v) CHAPS, 30 mM Tris, pH 8.5).

### DIGE labeling and SDS PAGE

For differential comparison of secretion patterns 5 μg of protein extracts per sample were labeled with CyDye Fluor minimal dyes (GE Healthcare) according to the manufacturer's instructions. Protein extracts of low toxin producing strains were labeled with Cy5, extracts of high toxin producing strains were labeled with Cy3. Afterwards samples of corresponding low and high toxic strains were pooled, reduced with Laemmli sample buffer containing 0,5 M DTT for 10 min on ice, and alkylated with 14% (w*/v*) iodoacetamide for 10 min at room temperature. Finally labeled protein extracts were separated by SDS PAGE using SE600 vertical electrophoresis system (Hoefer). Fluorescence images were scanned with a Typhoon 9400 imager (GE Healthcare).

### Statistical analyses

Mean values and standard deviations (SD) were calculated from at least three independent experiments each. For statistical analysis, 2-tailed Student's *t* test was used, where applicable, to determine statistically significant differences by Microsoft Excel. Graphs of raw data were plotted using Microsoft Excel or GraphPad Prism (version 6.0). Spearman correlation was calculated using SigmaStat 3.5.

## Results

### Selection of the *B. cereus* strain set

For selection of the *B. cereus* strain set to be used in this study, 136 isolates from the strain collections of our institutes were characterized according to their genetic toxin profile. Additionally, they were classified to be of high, medium or low toxicity according to their cytotoxicity in routinely performed WST-1 bioassays on Vero cells and their production of enterotoxin component NheB (Table S1). 19 of these strains were selected for genome sequencing (Böhm et al., in preparation) and growth, enterotoxin gene transcription, enterotoxin production, protein secretion, and cytotoxicity were studied. The genetic relationship of the strains is depicted in Figure S1. The strains were of different origins (food or food poisoning outbreaks), of different genetic toxin profiles and of either high or low toxic activity (Table 1). Strains involved in food poisoning outbreaks were isolated either from the contaminated food or from patient feces. Also, the presence of *hlyII, inhA1*, and *nprA* was considered. Originally, equal numbers of strains per toxin profile were chosen, later two isolates from recent food poisoning outbreaks in Austria and England were added to profile A.

### Strong genetic conservation of enterotoxin components

Whole genome sequences of all 19 *B. cereus* strains were obtained using a *de novo next generation sequencing* approach (MiSeq®, Illumina®) (Böhm et al., in preparation). Enterotoxin genes *nheABC* and *hblCDA* were identified via BLASTN analysis using published *nhe* and *hbl* operon sequences from the reference strains NVH 0075-95 and F837/76 as template. Alignments of the concatenated operon genes using CloneManager 7 showed that nucleotide sequences of both enterotoxin operons are highly conserved over all *B. cereus* strains investigated. Similarity of *nheABC* genes ranges from 93 to 99%, while *hblCDA* genes are even more conserved (95–98%). In contrast to all other *hbl* encoding strains of the set that harbor the major *hblCDAB* operon, *B. cereus* MHI226 solely possesses the *hbl_a_* gene variant (Beecher and Wong, 2000) with a sequence similarity of 80–81% to the other *hblCDA* genes. *B. cereus* MHI226 does not form a functional Hbl toxin (data not shown) and was therefore reclassified in toxin profile F (only encoding *nheABC*). *Hbl_a_* (*hblCDA*_*a*_) is present in addition to the major toxin operon *hblCDAB* in about one third of all *hbl*-encoding strains (Böhm et al., in preparation), as it appears in strains 14294-3 (M6), 6/27/S and RIVM BC 126. Similarity between *hbl* and *hbl_a_* variants ranges from 75 to 82%.

As a consequence of the pronounced enterotoxin gene conservation, very high sequence identity can be observed in multiple amino acid (aa) sequence alignments for all enterotoxin components (Figures S2, S3). NheB is the most conserved Nhe component with 15 variable out of 402 aa (3.73%) (Figure S2B), compared to 35 out of 386 aa (9.07%) in NheA (Figure S2A) and 47 out of 359 aa (13.09%) in NheC (Figure S2C). Hbl L2 exhibits 37 variable out of 439 aa (8.43%) (Figure S3A), Hbl B 24 out of 375 (6.40%) (Figure S3C) and the highly conserved Hbl L1 6 out of 406 (1.48%) (Figure S3B).

Additionally, amino acid sequences of the putative virulence factors HlyII, InhA1, NprA, and sphingomyelinase (Cadot et al., 2010; Tran et al., 2011; Doll et al., 2013) were compared. As for Nhe, all 19 strains possess the genes for InhA1, NprA and SMase, while only 21.1% (4 out of 19, among them two high and two low toxic strains) bear *hlyII* (Table 1). Thus, *hlyII* was excluded from further investigations. The low toxic strain MHI226 even possesses two *hlyII* genes. In comparison to the enterotoxins, InhA1, NprA and SMase are less conserved harboring 11.3%, 9.0% and 16.2% variable aa positions, respectively (data not shown). Neither comparison of toxin or virulence factor genes, nor amino acid sequences displayed discriminatory differences between high and low toxic *B. cereus* strains or showed any clustering according to their origin (food, food poisoning, or feces). These results show that neither presence or absence nor gene and protein sequences of enterotoxin and virulence factors provide a basis to assess virulence of a *B. cereus* strain. Therefore, it may be suspected that differential toxicity may be due to regulatory processes at the transcriptional or translational level of toxin expression.

### Highly similar growth kinetics within the *B. cereus* strain set

**Figure 1:**
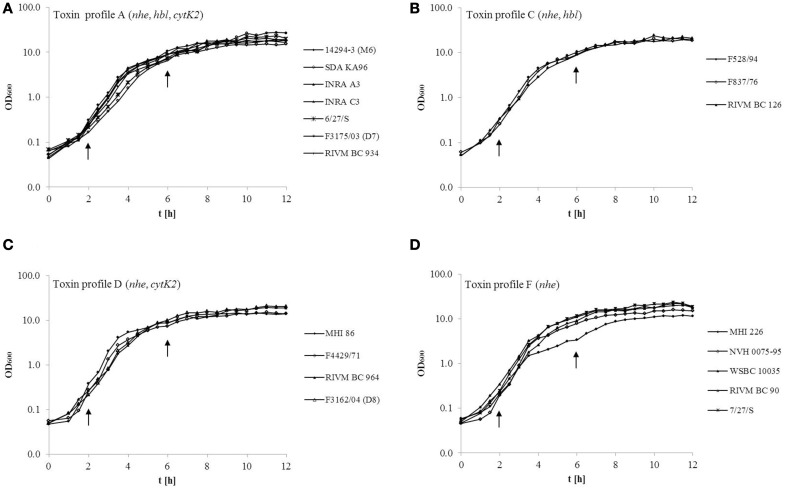
**Growth curves of 19 *B. cereus* strains grouped according to their toxin profile. (A)** toxin profile A (*nhe, hbl, cytK*), (**B**) toxin profile C (*nhe, hbl*), (**C**) toxin profile D (*nhe, cytK*), (**D**) toxin profile F (*nhe*). Strains were grown in CGY medium at 30°C, 125 rpm. Arrows indicate the time were samples were taken.

For comparative analyses of toxin transcription, production and secretion, all 19 *B. cereus* strains were grown under standard laboratory conditions. In Figure 1, the growth kinetics of strains grouped according to their toxin profile are shown. In general, growth kinetics of all *B. cereus* strains were very similar except for the low toxicity strain MHI226, which had a more shallow slope and reached the lowest maximal optical density of OD_600_ 11.9, while OD_600max._ of all other stains in toxin profile F ranged between OD_600_ 15.8–23.2 (Figure 1D). Maximal OD_600_ in toxin profile A (Figure 1A) was between OD_600_ 15.2–27.4, between OD_600_ 20.0–23.7 in toxin profile C (Figure 1B) and between OD_600_ 14.6–20.8 in toxin profile D (Figure 1C). Strains isolated from human feces (6/27/S, F3175/03 (D7), RIVM BC 126, F3162/04 (D8), RIVM BC 90, 7/27/S) tended to grow to higher maximal optical densities (OD_600_ > 20.0) than non-feces strains of the same toxin profile. However, significant differences in growth kinetics were only seen for MHI226. To account for any growth differences, further data were normalized to the optical density of the respective *B. cereus* strain at the time point analyzed.

### Strain-dependent toxin gene transcription

**Figure 2:**
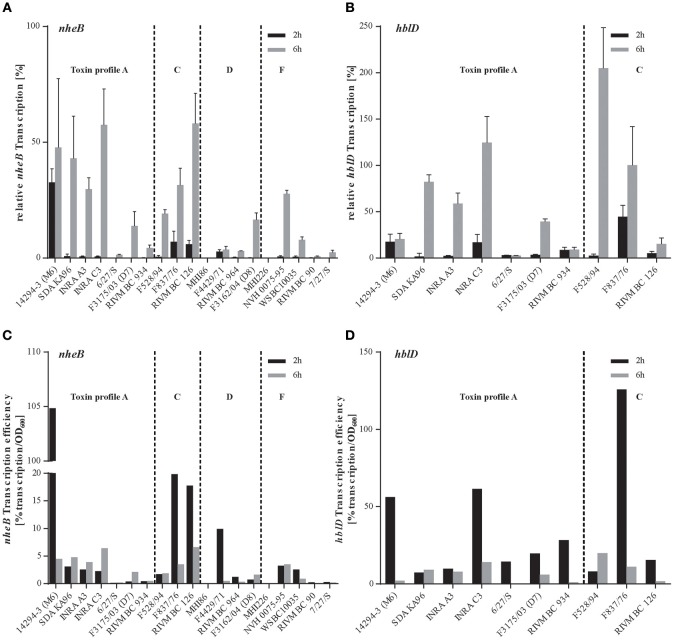
**Analyses of relative toxin gene transcription and transcription efficiency of the *B. cereus* strain set using qRT-PCR and the 2^-ΔΔC^_T_ method**. Total RNA was purified from 19 *B. cereus* strains harvested 2 (black) and 6 (gray) h after inoculation in CGY medium. Levels of *nheB* **(A)** and *hblD* **(B)** transcript were determined by qRT-PCR, normalized to 16S *rrn* levels of the same sample and relative to the transcript level of an external calibrator. The expression level of *hblD* of the toxin reference strain *B. cereus* F837/76 at 6 h served as calibrator that was set to 100% (log-2 = 0), all other transcript levels (A and B) were compared to this condition using the 2^-ΔΔ*C*_T_^ method (Livak and Schmittgen, 2001). Transcription efficiency was calculated as the ratio of mean relative transcript level of *nheB* **(C)** and *hblD* **(D)** in % per mean optical density of each strain (compare Figure 1).

Transcription of enterotoxin genes was analyzed using qRT-PCR. As previously described, *B. cereus* cytotoxicity strongly correlates with NheB and Hbl L1 concentrations in the supernatant (Jeßberger et al., 2014). Therefore, transcript levels (Livak and Schmittgen, 2001) of the corresponding genes *nheB* and *hblD* were determined. Relative *nheB* and *hblD* transcription [%] of all 19 *B. cereus* strains is presented in Figures 2A,B, respectively. In general, relative toxin gene transcription increased from 2 to 6 h in CGY medium, except for *B. cereus* 14294-3 (M6). Relative *hblD* transcription was higher than *nheB* transcription, except for strains 14294-3 (M6), RIVM BC 934 and RIVM BC 126 which showed 2.4 and 3.9-fold increase of *nheB* over *hblD* transcription in 6 h cultures. Unexpectedly, maximum *hblD* transcription occurred in F528/94 (Figure 2B), a strain classified as low toxic in the cytotoxicity assay. Relative transcription was further normalized to the OD_600_ and resulting transcription efficiencies (% transcription/OD_600_) are depicted in Figures 2C,D. In contrast to relative toxin transcript levels, toxin transcription efficiency did not uniformly increase from 2 to 6 h for all *B. cereus* strains, but showed strain-dependent kinetics. Eight strains of toxin profile A, C, D and F and of various origins showed decreased *nheB* transcription efficiency in 6 h cultures compared to 2 h, six strains had increased efficiency and five were similar at both growth stages (Figure 2C). In contrast, 80% of strains expressing Hbl showed higher *hblD* transcription efficiency at 2 h compared to 6 h except for SDA KA96 and F528/94 (Figure 2D). Interestingly, maximal *nheB* transcription efficiency was observed in the low toxic strain 14294-3 (M6) after 2 h in CGY medium, followed by the high toxic strains F837/76, RIVM BC 126 and F4429/71 (Figure 2C). Highest *hblD* transcription efficiency was achieved in 2 h cultures by F837/76, followed by INRA C3 and 14294-3 (M6) (Figure 2D).

In principle, toxin transcript levels of both *nheB* and *hblD* reflect the classification into high and low toxin producing strains according to their cytotoxicity (Table 1) to a certain extent. However, this is not generally the case. Setting a random threshold of 20% relative *nheB* and 40% *hblD* transcription for strains showing high toxin transcript levels, high cytotoxic strains F3175/03 (D7), F4429/71, F3162/04 (D8), WSBC10035, and 7/27/S cannot be considered to transcribe high levels of *nheB*, neither RIVM BC 126 for *hblD*. Moreover, some strains, e.g., 14294-3 (M6) are low toxic even though toxin gene transcription efficiency is amongst the highest, indicating that posttranscriptional and/or posttranslational regulation plays an important role for the manifestation of *B. cereus* toxicity. Furthermore, transcription of *inhA1, sph* and *nprA*, discussed as additional virulence contributors, proved to be highly strain-dependent (data not shown). In summary, there was no correlation of a strain's origin or genetic toxin profile and its toxin or virulence factor transcript level.

### Strain-specific enterotoxin production

**Figure 3:**
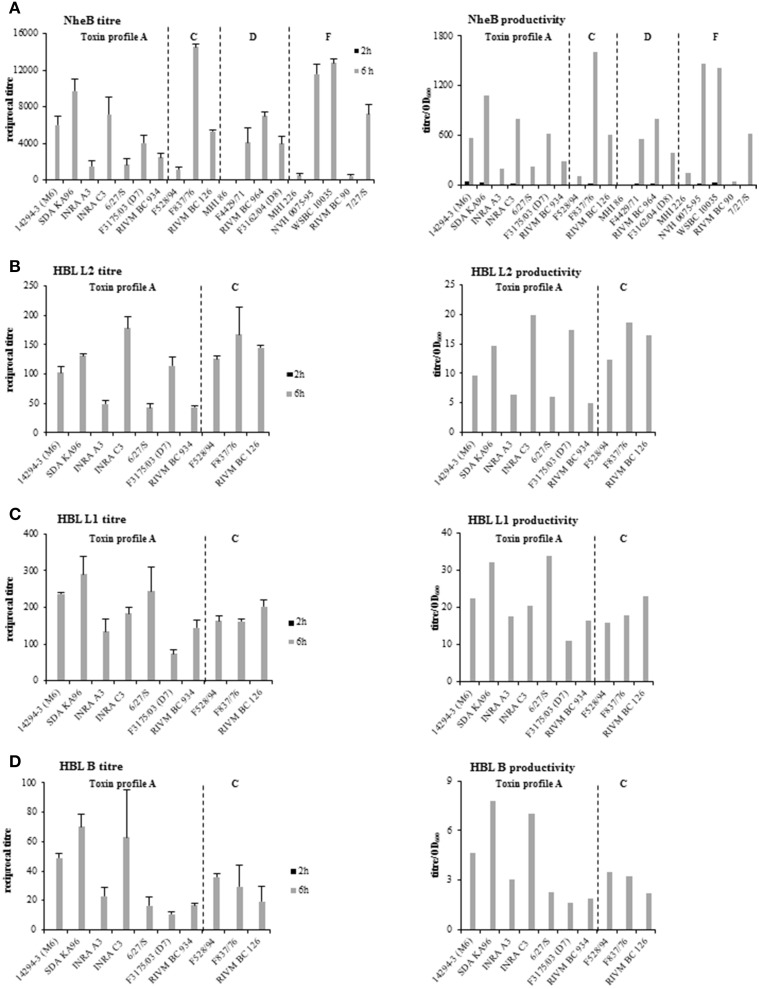
**Enterotoxin production of the *B. cereus* strain set as determined by sandwich and indirect EIAs**. Reciprocal titres as well as the productivity (titre/OD_600_) are shown. **(A)** NheB. **(B)** Hbl L2. **(C)** Hbl L1. **(D)** Hbl B.

Reciprocal titers of the enterotoxin components NheB, Hbl L2, Hbl L1, and Hbl B in the culture supernatants were determined performing specific EIAs. As a strong correlation of NheB with cytotoxicity has already been demonstrated (Moravek et al., 2006; Jeßberger et al., 2014), titers of NheA or NheC were not investigated. The productivity for all toxin components was calculated as titer/OD_600_ to exclude growth effects (Figure 3). After 2 h, no Hbl and only trace amounts of NheB protein were detectable. After 6 h, NheB titers generally confirmed prior classification into high, medium and low toxin producing strains (compare Table 1). Only 14294-3 (M6) showed higher NheB levels than in previous experiments (Figure 3A). Strains producing high, medium and low amounts of NheB were found among all toxin profiles as well as among food- and food poisoning-associated strains. NheB titers of strain F3162/04 (D8) were determined using only NheB-specific mab 1E11, as this strain is negative in sandwich EIA (A. Didier, in preparation). As the indirect EIA is not as sensitive as the sandwich, NheB production of this strain most likely is higher than detected with our tools.

The toxin pattern of the three Hbl components was similar to Nhe, i.e., strains earlier classified as *high* showed high amounts of all Hbl components, *low* strains showed lower Hbl production (Figures 3B–D). Interestingly, the two strains 6/27/S and F3175/03 (D7) showed similar toxic activity (Table 1 and **Figure 6**), but a different pattern. While 6/27/S produced comparably high amounts of Hbl L1 but low amounts of L2 and B, F3175/03 (D7) showed a comparably high titer and productivity for Hbl L2 but low L1 and B. Strain F528/94 showed similar Hbl titers compared to strain F837/76, though classified as a pair of low and high toxicity strains of the same genetic toxin profile (Table 1), suggesting that the total toxic activity as determined by WST-1-bioassays on Vero cells depends on Nhe rather than Hbl production. As for NheB, no specific pattern of Hbl production was found among the toxin profiles or food and food poisoning strains. NheB and Hbl L1 titers were compared to the transcription levels of their corresponding genes *nheB* and *hblD* using the Spearman correlation test. Significant dependence (*P* values < 0.05) of secreted NheB on relative *nheB* transcript level could be demonstrated for all time points investigated. Spearman correlation coefficient R was 0.817 (2 vs. 2 h), 0.605 (6 vs. 6 h) and 0.600 for comparing 2 h transcription with 6 h toxin titers. Poor influence (*P* > 0.05) of *hblD* transcription on Hbl L1 titer was detected.

### Comparable protein secretion ability

**Figure 4:**
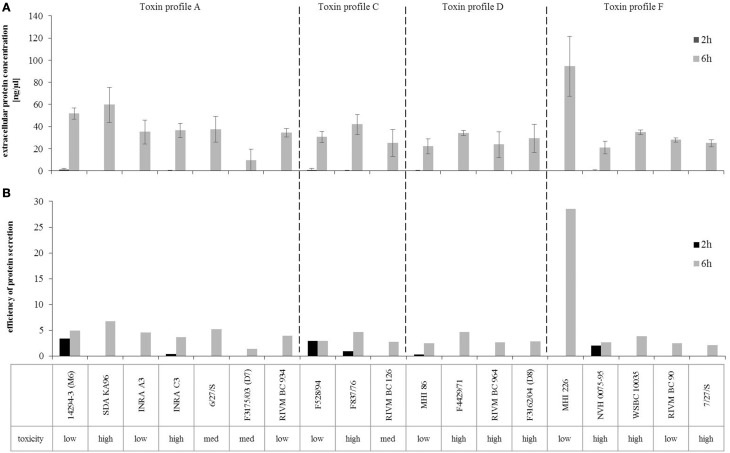
**Extracellular protein concentrations and secretion efficiency of the ***B. cereus*** strain set. (A)** Extracellular protein was quantified 2 and 6 h after inoculation. **(B)** To determine the efficiency of protein secretion, extracellular protein concentrations were normalized to the optical density (OD_600_) of the strains.

To analyze whether enterotoxin production generally correlates with the amount of secreted proteins, total extra- and intracellular protein concentrations were quantified. Intracellular protein concentrations were similar among all isolates tested (data not shown). Extracellular protein contributed to up to 10% of total protein content. 2 h after inoculation, only few extracellular protein concentrations were detectable (Figure 4A), while extracellular proteins of about 20–60 ng/μl were isolated after 6 h of growth under the standard laboratory conditions. For MHI226 total extracellular protein concentrations of 90 ng/μl were found. Extracellular protein content was normalized to the optical density of the strains to calculate the efficiency of protein secretion. As depicted in Figure 4B, all strains, except MHI226, showed comparable values. Generally, secretion efficiency was higher after 6 h of growth than at early growth phase. Only isolates 14294-3 (M6), F528/94 and NVH0075-95 revealed comparable efficiency values at 2 and 6 h. However, no significant differences in extracellular protein concentrations or efficiency of protein secretion were found between low and high toxic strains.

### Exoprotein profiling by DIGE analysis

**Figure 5:**
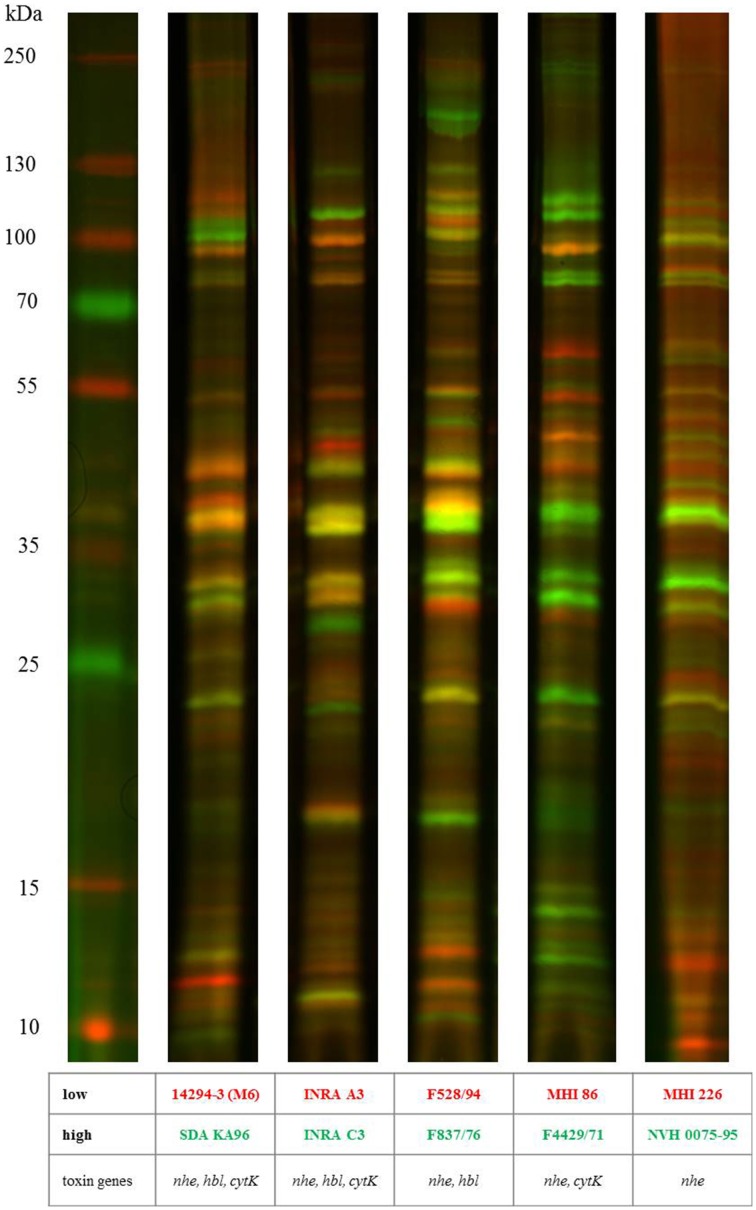
**1D DIGE exoprotein profiling of selected *B. cereus* isolates**. Exoproteins were isolated 6 h after inoculation and precipitated by trichloroacetic acid overnight. Five microgram of protein extracts were labeled with CyDye Fluor minimal dyes and separated by SDS PAGE. Proteins present in low toxin producing strains are shown in red (Cy5), proteins present in high toxin producing strains are displayed in green (Cy3). Similar protein levels are indicated by yellow bands.

To study secretion patterns of selected *B. cereus* isolates, secreted proteins were isolated 6 h after inoculation and analyzed by SDS-PAGE. Extracellular protein extracts of high and low toxin producing strains of the same genetic toxin profile were labeled with fluorescent Cyanine Dyes and separated in one single lane. Analysis of exoprotein profiles revealed distinct differences between the *B. cereus* isolates (Figure 5). Various proteins were differentially regulated in high and low toxic strains. However, secretion patterns of *B. cereus* isolates show high variability between the strains and no marker bands specific for high or low toxic strains could be detected by DIGE analysis. Silver staining of SDS-PAGE analyzed exoprotein extracts of the remaining 9 strains confirmed the high variability of secretion patterns (data not shown).

### Strain-specific cytotoxic activity

**Figure 6:**
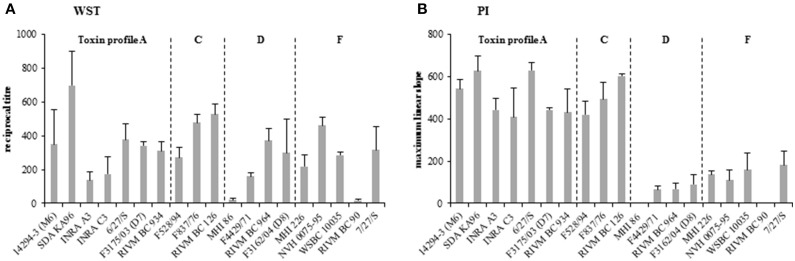
**Analysis of the toxic potential of the *B. cereus* strain set. (A)** Results of a WST-1 bioassay are shown as reciprocal titres. (**B**) Results of a propidium iodide influx test. The maximum linear slope of the fluorescence curve of each strain is shown. As after 2 h of bacterial growth no cytotoxicity was detected, only the toxicity after 6 h of growth is shown.

Cytotoxicity assays were performed using the human colon carcinoma cell line CaCo-2 instead of the routinely used Vero cells. As a consequence, the classification as high, medium or low toxic (Table 1) was altered. 14294-3 (M6), RIVM BC 934 and MHI226, former low toxic, now showed relatively high cytotoxicity in comparison with all strains. On the other hand, for INRA C3 and F4429/71, former highly toxic strains, comparably low cytotoxicity titers were obtained (Figure 6A). Of special interest were the strains F837/76 and F528/94 (high and low toxic, sharing the same genetic enterotoxin profile). Although F837/76 showed 12 x higher NheB production than F528/94 (Figure 3A), host cell toxicity was only 1.8 x increased (Figure 6A). Hbl titers were similar in both strains (Figures 3B–D), underlining that CaCo-2 cells are more sensitive to Hbl than Nhe. Our data indicate that results of the WST-1 bioassay depend very much on the target cell line and that for studying the toxic potential of enteropathogenic *B. cereus* in the consumer human colon cell lines such as CaCo-2 must strictly be used to avoid underestimation of certain strains.

Additionally, propidium iodide influx tests were performed to determine the speed of pore formation by the enterotoxins. Strains were compared according to the speed of PI influx shown as the maximum linear slope of the fluorescence curves (Figure 6B). Compared to the WST-1 bioassay, almost no differences between high and low toxic strains appeared, whereas strains producing Nhe and Hbl caused a significantly faster PI influx than solely Nhe producing strains. This phenomenon, as observed before (Jeßberger et al., 2014), suggests that pore formation in CaCo-2 cell membranes is triggered by the presence of Hbl and additionally that even low amounts of Hbl cause rapid PI influx.

## Discussion

So far, the reasons why *B. cereus* strains with identical genetic toxin profile produce such variable amounts of enterotoxins are not understood, i.e., it is yet to be investigated at which levels the varying pathogenic potential originates and is regulated. Moreover, it is unclear, whether further markers (proteins or nucleotides) can be identified for the differentiation between high and low toxic strains. To our knowledge, the presented study is the first comprehensive analysis of a representative set of enteropathogenic *B. cereus* strains that integrates many levels of toxin formation, from gene sequence to host cell cytotoxicity.

### Strain set

Currently, the most reliable method for predicting the toxic potential of enteropathogenic *B. cereus* is the quantification of the secreted enterotoxins (Guinebretière et al., 2002; Moravek et al., 2006). This can also be seen in our strain set, which can be divided into “pairs” of high and low toxic strains with identical genetic background. For example, INRA A3 and INRA C3, both clade II, toxin profile A, *hlyII−, inhA1+, nprA+*, share over 99% and 98% sequence similarity for Nhe and Hbl, but low vs. high amounts of the enterotoxin components are found in the respective culture supernatant. This was also observed for F528/94 (low) and F837/76 (high), both clade I, toxin profile C, *hlyII+, inhA1+, nprA+*, with 96% and 97% sequence similarity for Nhe and Hbl, respectively. The Nhe toxin components of MHI86 (low) and F4429/71 (high) (clade I, profile D, *hlyII−, inhA1+, nprA+*) even were 100% identical. So far, no connection between a certain enterotoxin gene profile, i.e., the presence of certain enterotoxin genes, and the toxic potential was found (Ceuppens et al., 2013). Even the presence of the PlcR/PapR regulatory system is no reliable indication for toxic activity (Fagerlund et al., 2007). Our data suggest that cytotoxicity of *B. cereus* is rather independent of toxin gene presence and sequence polymorphisms.

### Enterotoxin gene transcription

In a first systematic approach we compared enterotoxin gene expression of a representative *B. cereus* strain set. Previous studies compared enterotoxin expression in single *B. cereus* strains under varying growth temperature (Van Netten et al., 1990; Fermanian et al., 1997; Rejasse et al., 2012), oxygen availability (Duport et al., 2004; Van Der Voort and Abee, 2009), lowered oxidation-reduction potential (ORP) (Duport et al., 2006; Zigha et al., 2007; Esbelin et al., 2009) and differing availability of nutrients, for instance sugars (Ouhib et al., 2006; Ouhib-Jacobs et al., 2009). In our study, quantification of *nheB* and *hblD* mRNA levels at early exponential and late exponential growth phase/transition phase by qRT-PCR revealed highly strain-dependent variations in toxin gene transcript levels and transcription efficiency. It was further shown that toxin production is only to a certain extent determined by the level of enterotoxin transcription, which suggests that further posttranscriptional and posttranslational processes are involved.

The global virulence regulator PlcR strongly induces transcription of all enterotoxin genes during entry into the stationary growth phase (Gohar et al., 2008). PlcR-driven toxin gene expression might continue until *B. cereus* faces nutrient limitation at late stationary growth and undergoes sporulation, while at the same time *plcR* transcription is repressed by the transition state regulator Spo0A (Lereclus et al., 2000). However, in the present study transcription of *nheB* and *hblD* could be detected as early as 2 h after inoculation, and increased markedly during exponential growth until 6 h. These results coincide with the findings of Frenzel et al. (2012) on high *plcR* transcript levels during early *B. cereus* growth phase and reemphasize the direct positive transcriptional regulation of the enterotoxin operons by PlcR (Frenzel et al., 2012).

Comparison of the PlcR regulator proteins of our strain set revealed minor differences, especially in the less conserved C-terminal part of the protein (data not shown), which is also truncated in *B. anthracis* (Agaisse et al., 1999; Gohar et al., 2005). With the exception of one strain, PlcR of all *B. cereus* strains investigated shared between 87 and 100% similarity. Transcription analysis of the *plcR* regulator gene for a subset of *B. cereus* strains resulted in less pronounced strain-specificity compared to toxin gene transcription, but demonstrated similar *plcR* transcription kinetics for toxin profile A/C and D/F. Generally, no enhanced *plcR* transcript levels could be measured for *B. cereus* isolates classified as high in contrast to low toxic strains, except for the pair MHI86 (low) and F4429/71 (high) (Figure S4). Furthermore, no correlation between *plcR* transcription and *nheB* or *hblD* transcript levels could be determined. However, another pattern for differentiation of isolates was found: all four strains of toxin profile A and C showed increased *plcR* transcription after 6 h compared to 2 h, while the remaining six strains of toxin profile D and F transcribed lower amounts of *plcR* and tend to decrease *plcR* transcription at the later growth stage (Figure S4).

The present study focused on the comparison of a *B. cereus* strain set under identical aerobic culture conditions. Hence, induction of environment-sensing regulator proteins Fnr, ResDE, CcpA or CodY (Duport et al., 2006; Zigha et al., 2007; Van Der Voort et al., 2008; Esbelin et al., 2009, 2012; Messaoudi et al., 2010) was assumed to be comparable between the strains, their impact on differential toxin gene expression being negligible. Furthermore, since Fnr, ResDE, CcpA, and CodY protein sequences are highly conserved (sequence similarity > 98% and identity > 96%, data nor shown), variations in functionality are unlikely to account for strain-specific differences in toxin gene activation. Also, comparison of the *nheABC* and *hblCDAB* operon promoter regions of the strain set revealed only minor DNA sequence differences between essential promoter elements and regulator binding sites, precluding mutations that could explain single strain *nheB* or *hblD* transcript levels (data not shown).

Enterotoxin gene transcription only partially reflects our isolates' actual toxicity, indicating posttranscriptional and/or translational regulation. Alternative regulation of gene expression by non-coding RNA molecules is well-known in bacteria. RNAs might act as antisense RNA or form alternative structures, so called riboswitches, that promote or inhibit target gene translation in response to small molecule binding or temperature changes (for review see Johansson and Cossart, 2003; Batey, 2006). It has been demonstrated that several virulence determinants of the Gram-positive pathogen *Listeria monocytogenes* are controlled posttranscriptionally by long 5′ untranslated RNAs (Wong et al., 2004; Shen and Higgins, 2005; Loh et al., 2006). Involvement of stabilized mRNA transcripts in the regulation of *cryIIIA* toxin gene expression has been shown in *B. thuringiensis* and more recently gene control by riboswitches has been characterized in *B. anthracis* and *B. subtilis* (Welz and Breaker, 2007; Irnov et al., 2010; Wilson-Mitchell et al., 2012). The observation of unusually long 5′ untranslated regions (UTR) upstream of the start codons of both enterotoxin operons that span over 600 bp for P*_hbl_* and 300 bp for P*_nhe_* (data not shown; see also Agaisse et al., 1999) may point to a role in posttranscriptional regulation via formation of regulatory mRNA structures. Hence, deciphering a possible function of the long 5′ UTRs in enterotoxin expression of *B. cereus* is subject to ongoing investigations.

### Protein secretion

All 19 *B. cereus* strains included in this study were capable of significant protein secretion. Under laboratory growth conditions all strains showed comparable extracellular protein concentrations and no differentiation between high and low toxicity strains were obvious. Thus, a defect in general protein secretion can be excluded as a potential cause for the low toxicity of certain strains.

*B. cereus* enterotoxins are most likely secreted via the *sec* translocation pathway (Tjalsma et al., 2004; Fagerlund et al., 2010; Vörös et al., 2014). Complete amino acid sequence comparisons performed in our study showed that Sec-type secretion signal peptides of all Nhe and Hbl enterotoxin components, as predicted using the SignalP method, are highly conserved, for NheB and Hbl L1 even up to 100% (Figures S2, S3). Especially, signal peptidase cleavage sites are identical with the exception of NheA, where 14294-3 (M6) showed an exchange of the basic amino acid lysine against the negatively charged, hydrophilic amino acid residue glutamate K27E (Figure S2A). It has already been shown that a modification of the signal peptide sequence of Hbl B within the hydrophobic region leads to loss of secretion and intracellular accumulation of the protein (Fagerlund et al., 2010). In our study, most amino acid exchanges could be found in the signal peptide sequence of NheC and Hbl B. While most changes are neutral for the charge of the signal peptide sequences of Nhe and Hbl, some substitutions of the neutral alanine residue to threonine render the hydrophobic region more hydrophilic: A19T (NheB), A12T (NheC), A21T (Hbl B) (Figures S2B, S2C, S3C). However, no correlation of signal peptide mutations with high and low toxicity was found. Only the signal peptide sequence of Hbl B in the highly toxic isolate RIVM BC 126 showed several amino acid residue substitutions that might explain why high levels of Hbl L1 and Hbl L2 were detected in the secretome of RIVM BC 126, but only relatively low amounts of Hbl B (Figure 3).

Additionally, we compared extra- and intracellular amounts of NheB and Hbl L1 to exclude potential defects in the secretory mechanisms of our strains. Though taken in an independent approach, NheB and Hbl L1 titers in the culture supernatants were comparable to those in Figure 3. No or only trace amounts of NheB and Hbl L1 were detectable in the cytoplasm (data not shown), suggesting that the enterotoxins of all strains are secreted immediately and effectively after translation. Furthermore, intracellular titers rose in comparison to extracellular titers after treatment of the strains with sodium azide (data not shown), which inhibits the ATPase function of SecA (according to Fagerlund et al., 2010). This indicates that the enterotoxins of all 19 strains are indeed secreted via a fully functional Sec translocation pathway.

Besides enterotoxins, *B. cereus* secretes a variety of other proteins such as proteases, phospholipases and other members of the PlcR regulon (Gohar et al., 2008). For *B. anthracis* it has been shown that own bacterial proteases contribute to virulence by directly degrading anthrax toxins or modulating concentrations and activity of other extracellular proteases (Pflughoeft et al., 2014). The proteases InhA1 and NprX can represent up to 90% of the bacterium's secretome (Chitlaru et al., 2006). The concentrations of the respective proteins in the secretome of the *B. cereus* strain set cannot be detected so far, as suitable detection methods (e.g., EIAs based on specific antibodies) still have to be developed. We suppose it may rather be possible that the low toxic strains generally secrete higher levels of proteases, which subsequently degrade the secreted enterotoxins, than secreting low amounts of enterotoxins and proteases. Enterotoxin gene expression of strain MHI226 was hardly detectable, but cytotoxic activity on CaCo-2 cells was comparably high. Perhaps, the lack of enterotoxin production was compensated by enhanced protein secretion. Generally, MHI226 showed quite extraordinary properties concerning growth, enterotoxin gene expression, toxicity and especially protein secretion, thus it might be a suitable candidate to further study the relationship of protein secretion and toxicity in *B. cereus*.

### Enterotoxin production and toxicity

Enterotoxin titers generally confirmed the previous classification of high, medium and low toxin producing strains. Comparison of F3162/04 (D8) with the other strains must be done with caution, as NheB titers were obtained in indirect EIA using only mab 1E11. In preliminary experiments, this strain showed low NheB production but in comparison to that unusually high cytotoxicity (Table 1, Table S1). Sequence analyses revealed a point mutation in the *nheB* gene resulting in the exchange of amino acid residue 151 from glutamic to aspartic acid. This residue is situated in direct proximity to the predicted epitope of mab 2B11, which is used in sandwich EIAs for NheB detection (Didier et al., 2012). This mutation might contribute to a loss of 2B11 binding capacity and thus, detection of the NheB protein, but without affecting its functionality (A. Didier, in preparation). During selection of our strain set, three strains (F3162/04 (D8), MHI 124 and HWW 274-1) out of 136 showed this phenomenon, implying that the toxic potential of 2.2% of all enteropathogenic *B. cereus* isolates might be underestimated when NheB production is investigated with sandwich EIAs based on mab 2B11.

Pearson correlation tests showed significant correlation of cytotoxicity, obtained with WST-1 bioassays on CaCo-2 cells, and NheB titers after 6 h of growth (correlation coefficient 0.61). The same WST test was performed on Vero cells (data not shown), with which NheB titers correlated even better (correlation coefficient 0.84). This confirmed prior observations that cytotoxicity depends significantly on NheB production and that Vero cells are even more sensitive, particularly toward Nhe and generally to *B. cereus* culture supernatants than CaCo-2 cells (Moravek et al., 2006; Jeßberger et al., 2014). Nevertheless, CaCo-2 cells should be used rather than Vero cells to investigate the cytotoxic activity, as *B. cereus* enterotoxins operate rather in the human intestine than in kidney. Furthermore, discrepancies between high and low toxic strains are bigger on Vero cells, which might lead to underestimation of putative non-pathogenic strains. Contrary to earlier studies (Jeßberger et al., 2014) no significant correlation between Hbl production and cytotoxicity was found, possibly due to the small number of strains investigated (10 *hbl* positive strains). We further observed that for uncovering the varying toxic potential of different *B. cereus* isolates the choice of cytotoxicity assay is very important. While differences between high and low toxic strains of our strain set became obvious in WST-1 bioassays, these differences were no longer detectable when PI influx tests were performed (Figure 6). The 10 strains expressing Nhe and Hbl showed comparably quick PI influx and thus pore formation while the 9 strains expressing only Nhe showed only poor PI influx. This supports the assumption that, although high sequence homologies between the Nhe and the Hbl components exist (Granum et al., 1999), the mode of action and the way of pore assembly and formation of these two enterotoxin complexes differs significantly.

## Conclusion

In summary, our data suggest that the highly variable toxic potential of *B. cereus* is determined by complex and dynamic regulatory processes involving toxin gene transcription strictly regulated by known regulator proteins as well as strain-specific posttranscriptional or posttranslational toxin modifications affecting mRNA stability, translation initiation or protein durability and, perhaps, resistance to extracellular proteolytic degradation. Furthermore, high and low toxic strains could not be correlated with certain groups such as food or food poisoning isolates or enterotoxin gene profiles. Also, the presence of genes encoding further putative virulence factors such as hemolysin II, sphingomyelinase or the metalloproteases InhA1 and NprA provided no further information to predict high toxicity in *B. cereus* strains.

Besides these intrinsic bacterial factors, the condition of the host (age, immunity, etc.) as well as the composition of the contaminated food may contribute to the high variability of enterotoxin production (Ceuppens et al., 2013), which makes the toxic potential even more difficult to assess and to predict. In this study, using standard laboratory growth conditions which are routinely applied for the assessment of *B. cereus* toxicity, no distinct bacterial factors or mechanisms responsible for the highly variable enterotoxin production and thus cytotoxic potential were found. Therefore, the question arises whether enterotoxin production in the intestine is triggered or activated by environmental factors and whether potentially high and low toxic strains respond differentially to such factors. We showed that the pattern of high and low toxic strains was already altered when an alternative target cell line was used in cytotoxicity assays (CaCo-2 instead of Vero). It may be speculated that this pattern is further influenced by growth of bacteria under simulated intestinal instead of standard laboratory conditions or in the presence of colon epithelial cells.

### Conflict of interest statement

The authors declare that the research was conducted in the absence of any commercial or financial relationships that could be construed as a potential conflict of interest.
