# Supplementary material for: From genome to toxicity: a combinatory approach highlights the complexity of enterotoxin production in Bacillus cereus
Source: Front Microbiol. 2015 Jun 10;6:560. doi: 10.3389/fmicb.2015.00560 (PMC4462024; doi:10.3389/fmicb.2015.00560)
Supplement: Supplementary file 1 [file Table1.DOCX]

**Tab. S1: Set of 136 *B. cereus* isolates characterized for this study.** Genotyping was performed by sequence analyses of the genetic markers *spoIIIAB* or *panC*, toxin profiling by PCR analyses using specific primers for *ces* and enterotoxin genes, toxin titers were determined by sandwich EIAs against NheB and toxicity was analyzed by WST-1-bioassay on Vero cells. Isolates were classified highly (hi), medium (m) or low (lo) toxic according to their cytotoxicity (hi: >500; m: 250-500; lo: <250) and NheB titers (hi: >4000; m: 2000-4000; lo: <2000).

| ***B. cereus* strain** | **origin** |  | **genotype**  **clade (group)** | **toxin gene profiling**  ***ces hbl nhe cytK2*  profile** | | | | | **toxin titers**  **NheB** | **toxicity**  **Vero** | |  |
| --- | --- | --- | --- | --- | --- | --- | --- | --- | --- | --- | --- | --- |
| WSBC10028 | past. milk | food | II (IV) | - | + | + | + | A | lo | | m | |
| INRA 32* | zucchini purée | food | II | - | + | + | + | A | lo | | hi | |
| NVH 0230-00 | mushroom stew, food poisoning outbreak | diarrheal | II (IV) | - | + | + | + | A | m | | hi | |
| NVH 1230/88* | mushroom stew | diarrheal | II (IV) | - | + | + | + | A | m | | m | |
| 98HMPL63 * | cooked salsify | diarrheal | II (IV) | - | + | + | + | A | m | | m | |
| INRA A3* | starch | food | II (IV) | - | + | + | + | A | lo | | lo | |
| INRA C3* | past. carrot | food | II (IV) | - | + | + | + | A | hi | | hi | |
| F2141/74 | no further information | diarrheal | II | - | + | + | + | A | m | | m | |
| SDA KA96* | raw milk | food | I (III) | - | + | + | + | A | hi | | hi | |
| F2081/98* | cooked chicken | diarrheal | I | - | + | + | + | A | m | | hi | |
| F3371/93* | chinese takeaway chop suey | diarrheal | II | - | + | + | + | A | lo | | m | |
| F352/90 | chow mein | diarrheal | II (IV) | - | + | + | + | A | m | | hi | |
| F4430/73* | pea soup | diarrheal; toxin reference | II (IV) | - | + | + | + | A | m | | m | |
| F4433/73* | meat loaf | diarrheal | II | - | + | + | + | A | lo | | m | |
| INRA I16* | past. potatoes | food | II (IV) | - | + | + | + | A | lo | | m | |
| IH41064* | faeces | diarrheal | II | - | + | + | + | A | lo | | m | |
| LMG 17615* | pork pie | diarrheal | II | - | + | + | + | A | m | | m | |
| WSBC10395 | raw milk | food | II (IV) | - | + | + | + | A | lo | | hi | |
| INRA C57* | potato purée | food | II (IV) | - | + | + | + | A | m | | hi | |
| MHI1757 | milk | food | n.d. | - | + | + | + | A | lo | | lo | |
| 95-Bw | food isolate | food | n.d. | - | + | + | + | A | hi | | hi | |
| 112-Bw | food isolate | food | n.d. | - | + | + | + | A | hi | | hi | |
| 135-Bw | kitchen surface | surface | n.d. | - | + | + | + | A | m | | m | |
| 144-Bw | kitchen surface | surface | n.d. | - | + | + | + | A | hi | | hi | |
| 156-Bw | kitchen surface | surface | n.d. | - | + | + | + | A | m | | m | |
| 171-Bw | food isolate | food | n.d. | - | + | + | + | A | hi | | hi | |
| 179-Bw | hands | surface | n.d. | - | + | + | + | A | m | | lo | |
| 14294-3 (M6) | ice cream | food | I | - | + | + | + | A | m | | m | |
| RIVM BC 118 | human faeces | diarrheal | II | - | + | + | + | A | m | | m | |
| F3175/03 (D7) | human faeces | diarrheal | II | - | + | + | + | A | hi | | m | |
| 2/27/S | human faeces | diarrheal | II (IV) | - | + | + | + | A | m | | m | |
| 6/27/S | human faeces | diarrheal | II (IV) | - | + | + | + | A | m | | m | |
| MHI236 | milk and milk products | food | III (VI) | *-* | *+* | *+* | *-* | C | lo | | lo | |
| INRA C1 | past. zucchini | food | III (VI) | - | + | + | - | C | m | | hi | |
| F837/76* | human, postoperative infection | Hbl reference | I (III) | - | + | + | - | C | hi | | hi | |
| F4370/75 | barbecue chicken | diarrheal | I (III) | - | + | + | - | C | lo | | hi | |
| INRA I21 | cooked carrot | food | III (VI) | - | + | + | - | C | lo | | lo | |
| INRA PF | milk proteins | food | I (III) | - | + | + | - | C | lo | | lo | |
| RIVM BC 934* | lettuce | food | II (IV) | - | + | + | + | A | lo | | lo | |
| 97-Bw | kitchen surface | surface | I | - | + | + | - | C | hi | | hi | |
| 98-Bw | kitchen surface | surface | I | - | + | + | - | C | hi | | me | |
| HW274-6b | infant food | food | I | - | + | + | - | C | m | | hi | |
| Routineiso R3 | milk powder | food | I | - | + | + | - | C | hi | | m | |
| Routineiso R4 | milk powder | food | I | - | + | + | - | C | hi | | m | |
| ESP11 | fast food slices | food | I | - | + | + | - | C | hi | | m | |
| F528/94 | beef & chow mein and rice, food  poisoning outbreak | diarrheal | I (II) | - | + | + | - | C | lo | | lo | |
| WSBC10926 | cauliflower | food poisoning | II (V) | - | + | + | - | C | lo | | lo | |
| NVH141/1-01 | vegetarian pasta | food borne outbreak | II (V) | - | + | + | - | C | lo | | lo | |
| F2769/77 | lobster pate | food borne outbreak | II (IV) | - | + | + | - | C | lo | | lo | |
| 1/21 AGES | cutlet roll | food | II (V) | - | + | + | - | C | m | | m | |
| 10/12/D-AGES | parsley | food | II | - | + | + | - | C | lo | | lo | |
| 18/12/S-AGES | parsley | food | II | - | + | + | - | C | lo | | lo | |
| 20/12/S-AGES | parsley | food | II | - | + | + | - | C | m | | lo | |
| 6/12/S-AGES | parsley | food | II | - | + | + | - | C | lo | | lo | |
| 15/12/S-AGES | parsley | food | II | - | + | + | - | C | lo | | lo | |
| 2/12/AR-AGES | parsley | food | II | - | + | + | - | C | lo | | lo | |
| 17/12/S-AGES | parsley | food | II | - | + | + | - | C | lo | | lo | |
| 23/12/S-AGES | parsley | food | II | - | + | + | - | C | lo | | lo | |
| RIVM BC 120 | human faeces | diarrheal | I (II) | - | + | + | - | C | hi | | m | |
| RIVM BC 126 | human faeces | diarrheal | I (II) | - | + | + | - | C | hi | | hi | |
| NVH200 | meat dish with rice | diarrheal | I (III) | - | + | + | - | C | hi | | m | |
| SDA GR285 | raw milk | food | III | - | + | + | - | C | n.d. | | n.d. | |
| PL10 | cream | food | II | - | + | + | - | C | m | | lo | |
| PL11 | cream | food | II | - | + | + | - | C | m | | lo | |
| 2/24 AGES | dried oregano | food | I (II) | - | + | + | - | C | n.d. | | n.d. | |
| 14177-3 (M14) | fresh milk | food | III | - | + | + | - | C | n.d. | | n.d. | |
| 15472-2 (M15) | fresh milk | food | III | - | + | + | - | C | n.d. | | n.d. | |
| MHI86 | infant food | food | I | - | - | + | + | D | lo | | lo | |
| NRS 404* | no further information | diarrheal | I (III) | - | - | + | + | D | m | | m | |
| F2085/98* | cooked rice | diarrheal | I (III) | - | - | + | + | D | hi | | m | |
| F4429/71 | vanilla pudding | diarrheal | I (III) | - | - | + | + | D | hi | | hi | |
| MHI124 | baby food | food | I (III) | - | - | + | + | D | lo | | m | |
| NVH0597-99* | mixed spices | diarrheal | I (III) | - | - | + | + | D | m | | hi | |
| 100-Bw | kitchen surface | surface | I | - | - | + | + | D | hi | | hi | |
| 110-Bw | food isolate | food | I | - | - | + | + | D | hi | | hi | |
| 127-Bw | food isolate | food | I | - | - | + | + | D | hi | | hi | |
| 134-Bw | food isolate | food | I | - | - | + | + | D | hi | | m | |
| 172-Bw | kitchen surface | surface | I | - | - | + | + | D | hi | | hi | |
| HW274-4a | infant food | food | I | - | - | + | + | D | m | | lo | |
| HWW274-2 | milk powder | food | I | - | - | + | + | D | m | | lo | |
| RIVM BC 964 | kebab | food | II (IV) | - | - | + | + | D | hi | | hi | |
| F3162/03 (D8) | stool | clinical | I | - | - | + | + | D | lo | | hi | |
| MHI24* | infant food | food | n.d. | - | - | + | n.d. | D/F | hi | | m | |
| MHI57* | infant food | food | n.d. | - | - | + | n.d. | D/F | hi | | m | |
| MHI61* | infant food | food | n.d. | - | - | + | n.d. | D/F | hi | | hi | |
| MHI71* | infant food | food | n.d. | - | - | + | n.d. | D/F | hi | | m | |
| MHI92* | infant food | food | n.d. | - | - | + | n.d. | D/F | hi | | m | |
| MHI147a | infant food | food | n.d. | - | - | + | n.d. | D/F | lo | | lo | |
| MHI112 | infant food | food | n.d. | - | - | + | n.d. | D/F | lo | | lo | |
| N 3 | infant food | food | n.d. | - | - | + | n.d. | D/F | lo | | lo | |
| Hi 14 | infant food | food | n.d. | - | - | + | n.d. | D/F | hi | | lo | |
| MHI126* | infant food | food | n.d. | - | - | + | n.d. | D/F | hi | | m | |
| MHI133 | infant food | food | n.d. | - | - | + | n.d. | D/F | lo | | lo | |
| WS 10201 | milk and milk products | food | III (VI) | - | - | + | n.d. | D/F | lo | | lo | |
| WS 10208 | milk and milk products | food | III (VI) | - | - | + | n.d. | D/F | lo | | lo | |
| WS 10210 | milk and milk products | food | n.d. | - | - | + | n.d. | D/F | lo | | lo | |
| S 74,7 | milk and milk products | food | n.d. | - | - | + | n.d. | D/F | lo | | lo | |
| MHI183* | milk and milk products | food | n.d. | - | - | + | n.d. | D/F | lo | | lo | |
| H-Milch 2 | milk and milk products | food | n.d. | - | - | + | n.d. | D/F | lo | | lo | |
| 5*5 | milk and milk products | food | n.d. | - | - | + | n.d. | D/F | lo | | lo | |
| 93-Bw | kitchen surface | surface | n.d. | + | - | + | - | E | n.d. | | hi | |
| UHDAM B102 | meat pie | food | I (III) | + | - | + | - | E | n.d. | | n.d. | |
| RIVM BC 938 | lamb's lettuce | food | I (II) | + | - | + | - | E | n.d. | | n.d. | |
| MHI105 | infant food | food | I | - | - | + | - | F | lo | | lo | |
| MHI194 | milk and milk products | food | I | - | - | + | - | F | lo | | lo | |
| MHI226 | milk and milk products | food | I | - | (-) | + | - | C/F | lo | | lo | |
| WSBC10035* | past. milk | food | II | - | - | + | - | F | hi | | hi | |
| NVH 0075-95* | stew with vegetables, food poisoning | Nhe reference | I (III) | - | - | + | - | F | hi | | hi | |
| NVH 0500-00* | potatoes in cream sauce, food poisoning outbreak | diarrheal | I (III) | - | - | + | - | F | hi | | hi | |
| NVH 1519-00 | stew with deer meat | diarrheal | I (III) | - | - | + | - | F | hi | | hi | |
| INRA C24* | past. carrot | food | I (III) | - | - | + | - | F | hi | | hi | |
| F3003/73* | no further information | diarrheal | I | - | - | + | - | F | m | | hi | |
| NVH506 | spices | food | outside (VII) | - | - | + | - | F | lo | | m | |
| 131-Bw | food isolate | food | I | - | - | + | - | F | hi | | hi | |
| 147-Bw | hand swap | surface | I | - | - | + | - | F | m | | lo | |
| 1440-1 (M1)* | butter cream | food | I | - | - | + | - | F | hi | | hi | |
| 2669-4 (M10)* | dried apricots | food | I | - | - | + | - | F | hi | | hi | |
| 3571 (M12)* | dessert | food | I | - | - | + | - | F | m | | hi | |
| HW274-3b* | infant food | food | I | - | - | + | - | F | hi | | hi | |
| HWW274-1 | milk powder | food | I | - | - | + | - | F | lo | | hi | |
| KM* | coconut milk | food | I | - | - | + | - | F | hi | | hi | |
| UHDAM B217 | human faeces | diarrheal | I (III) | - | - | + | - | F | hi | | hi | |
| F452/04 (D9) | human faeces | diarrheal | I | - | - | + | - | F | hi | | hi | |
| RIVM BC 63 | human faeces | diarrheal | I (III) | - | - | + | - | F | lo | | m | |
| RIVM BC 70 | human faeces | clinical | I (III) | - | - | + | - | F | lo | | m | |
| RIVM BC 90 | human faeces | diarrheal | I (III) | - | - | + | - | F | lo | | lo | |
| 1/25 AGES | stool | clinical | I | - | - | + | - | F | hi | | hi | |
| 2/25 AGES | human faeces | diarrheal | I (III) | - | - | + | - | F | hi | | hi | |
| 1/27/S | human faeces | diarrheal | I (III) | - | - | + | - | F | hi | | hi | |
| 3/27/S | human faeces | diarrheal | I (III) | - | - | + | - | F | hi | | hi | |
| 4/27/S | human faeces | diarrheal | I (III) | - | - | + | - | F | hi | | hi | |
| 5/27/S | human faeces | diarrheal | I (II) | - | - | + | - | F | lo | | lo | |
| 7/27/S | human faeces | diarrheal | I (III) | - | - | + | - | F | hi | | hi | |
| 2/7 AGES | mashed potatoes | food | I (III) | - | - | + | - | F | n.d. | | n.d. | |
| 13982-3 (M13) | fresh milk | food | III | - | - | + | - | F | n.d. | | n.d. | |
| NVH 0391-98* | vegetable pure | CytK reference | outside (VII) | - | - | - | + | G | lo | | lo | |

*: toxin and cytotoxicity titers of theses strains have previously been published (Jessberger et al., 2014).
