# Supplementary material for: From genome to toxicity: a combinatory approach highlights the complexity of enterotoxin production in Bacillus cereus
Source: Front Microbiol. 2015 Jun 10;6:560. doi: 10.3389/fmicb.2015.00560 (PMC4462024; doi:10.3389/fmicb.2015.00560)
Supplement: Supplementary file 2 [file Table2.DOCX]

**Tab. S2: Primers used in this study**

| **Primer** | **Target sequence** | **Sequence 5’ – 3’** | **Source** | | |
| --- | --- | --- | --- | --- | --- |
|  |  |  |  | | |
| **Primers for multiplex PCR** | |  |  |  |  |
|  |  |  |  | | |
| HD2 F | Fragment spanning *hblD-hblA* genes | GTA AAT TAI GAT GAI CAA TTTC | (Ehling-Schulz et al., 2006) | | |
| HA4 R |  | AGA ATA GGC ATT CAT AGA TT |  |  |  |
|  |  |  |  | | |
| NA2 F | Fragment spanning *nheA-nheB* genes | AAG CIG CTC TTC GIA TTC | (Ehling-Schulz et al., 2006) | | |
| NB1 R |  | ITI GTT GAA ATA AGC TGT GG |  |  |  |
|  |  |  |  | | |
| CK F2 | Inner fragment of *cytK* | ACA GAT ATC GGI CAA AAT GC | (Ehling-Schulz et al., 2006) | | |
| CK R5 |  | CAA GTI ACT TGA CCI GTT GC |  |  |  |
|  |  |  |  | | |
| CesF1 | Inner fragment of *ces* | GGTGACACATTATCATATAAGGTG | (Ehling-Schulz et al., 2005a) | | |
| CesR2 |  | GTAAGCGAACCTGTCTGTAACAACA |  |  |  |
|  |  |  |  | | |
| **Primers for qRT PCR** | |  |  |  |  |
|  |  |  |  | | |
| 16SA1 | Inner fragment of 16S rRNA gene *rrn* | GGAGGAAGGTGGGGATGACG | (Martineau et al., 1996) | | |
| 16SA2 |  | ATGGTGTGACGGGCGGTGTG |  |  |  |
|  |  |  |  | | |
| nheB_qRT_for | Inner fragment of *nheB* | GTGAAACAAGCTCCAGTTC | This work | | |
| nheB_qRT_rev |  | AAAGCGTACAGATCCATTACT |  |  |  |
|  |  |  |  | | |
| nheB_qRT_for2**^a^** | Inner fragment of *nheB*; used together with nheB_qRT_rev | GTGAAACAAGCTCCAATTCAT | This work | | |
|  |  |  |  | | |
| nheB_qRT_rev2**^b^** | Inner fragment of *nheB*; used together with nheB_qRT_for | AAAGCATACAGATCCATTACTAA | This work | | |
|  |  |  |  | | |
| hblD_qRT_for | Inner fragment of *hblD* | ATCTAAGCATATGGACTCCTA | This work | | |
| hblD_qRT_rev |  | CCATTGTATTCCATTGCTTTTG |  |  |  |
|  |  |  |  | | |
| hblD_qRT_for2**^c^** | Inner fragment of *nheB*; used together with hblD_qRT_rev | ATCTAAGCATATGGATTCCTATA | This work | | |
|  |  |  |  | | |
| **Primers for sequence typing** | |  |  |  |  |
| panC_for | Inner fragement of *panC* | CGA TAT CCT CGT GAT ATT GAT AGA G | (Candelon et al., 2004;Guinebretiere et al., 2008) | | |
| panC_rev |  | TCC GCA TAA TCT ACA GTG CCT TTC |  |  |  |
| Spo2F | Inner fragment of *spoIIIAB* | CGACGAGGATAACCCAATTTGC | (Ehling-Schulz et al., 2005a) | | |
| Spo2R |  | CAGTGAGAGACCGAGGCAAC |  |  |  |
|  |  |  |  | | |
| **^a^** In combination with nheB_qRT_rev used for amplification of an inner fragment of the *nheB* gene in *B. cereus* 14294-3 (M6), RIVM BC 126 and F3162/04 (D8) | | | | |  |
| **^b^** In combination with nheB_qRT_for amplification of an inner fragment of the *nheB* gene in *B. cereus* RIVM BC 964, RIVM BC 934, INRA A3, INRA C3, 6/27/S and F3175/03 (D7) | | | | |  |
| **^c^** In combination with hblD_qRT_rev amplification of an inner fragment of the *nheB* gene in *B. cereus* RIVM BC 934, INRA A3, 6/27/S and F3175/03 (D7) | | | | |  |
