# Supplementary material for: From genome to toxicity: a combinatory approach highlights the complexity of enterotoxin production in Bacillus cereus
Source: Front Microbiol. 2015 Jun 10;6:560. doi: 10.3389/fmicb.2015.00560 (PMC4462024; doi:10.3389/fmicb.2015.00560)
Supplement: Supplementary file 3 [file DataSheet1.DOCX]

Figure legends (supplementary figures)

**Fig. S1: Genetic relationship among the *B. cereus* strains used in this study, originated by *panC* sequence typing.** Cluster analysis and tree was constructed with TREECON software using the neighbour-joining method ([Van de Peer and De Wachter, 1997](#_ENREF_66)).

**Fig. S2: Multiple amino acid sequence alignments.** Enterotoxin components NheA **(A)**, NheB **(B)** and NheC **(C)** of 19 *B. cereus* strains were compared and aligned using Clustal Omega v1.2.0 ([Sievers et al., 2011](#_ENREF_61)). Signal peptide sequences were predicted using the SignalP 4.1 server ([Petersen et al., 2011](#_ENREF_56)). High toxin producing strains are highlighted in grey.

**Fig. S3: Multiple amino acid sequence alignments.** Enterotoxin components Hbl L2 **(A),** Hbl L1 **(B)** and Hbl B **(C)** of 10 Hbl producing *B. cereus* strains were compared and aligned using Clustal Omega v1.2.0 ([Sievers et al., 2011](#_ENREF_61)). Signal peptide sequences were predicted using the SignalP 4.1 server ([Petersen et al., 2011](#_ENREF_56)). High toxin producing strains are highlighted in grey.

**Fig. S4: Analysis of relative *plcR* gene transcription of 10 *B. cereus* strains using qRT-PCR and the 2^-ΔΔC^_T_ method.** Total RNA was purified from 10 *B. cereus* strains harvested 2 (black) and 6 (grey) h after inoculation. Levels of *plcR* transcript were normalized to 16S rrn levels of the same sample and relative to the transcript level of an external calibrator. The expression level of *hblD* of the toxin reference strain *B. cereus* F837/76 at 6 h (compare Fig. 2B) served as calibrator that was set to 100 % (log-2 = 0), all other transcript levels were compared to this condition using the 2^-ΔΔC^_T_ method ([Livak and Schmittgen, 2001](#_ENREF_44)).
