# Supplementary figures and images for: From genome to toxicity: a combinatory approach highlights the complexity of enterotoxin production in Bacillus cereus
Source: Front Microbiol. 2015 Jun 10;6:560. doi: 10.3389/fmicb.2015.00560 (PMC4462024; doi:10.3389/fmicb.2015.00560)

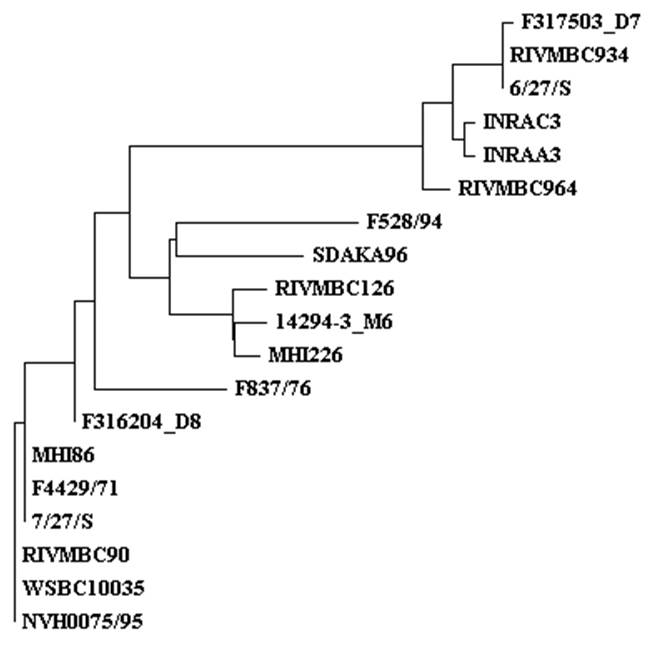

Supplement: Supplementary file 4 [file Image1.JPEG]

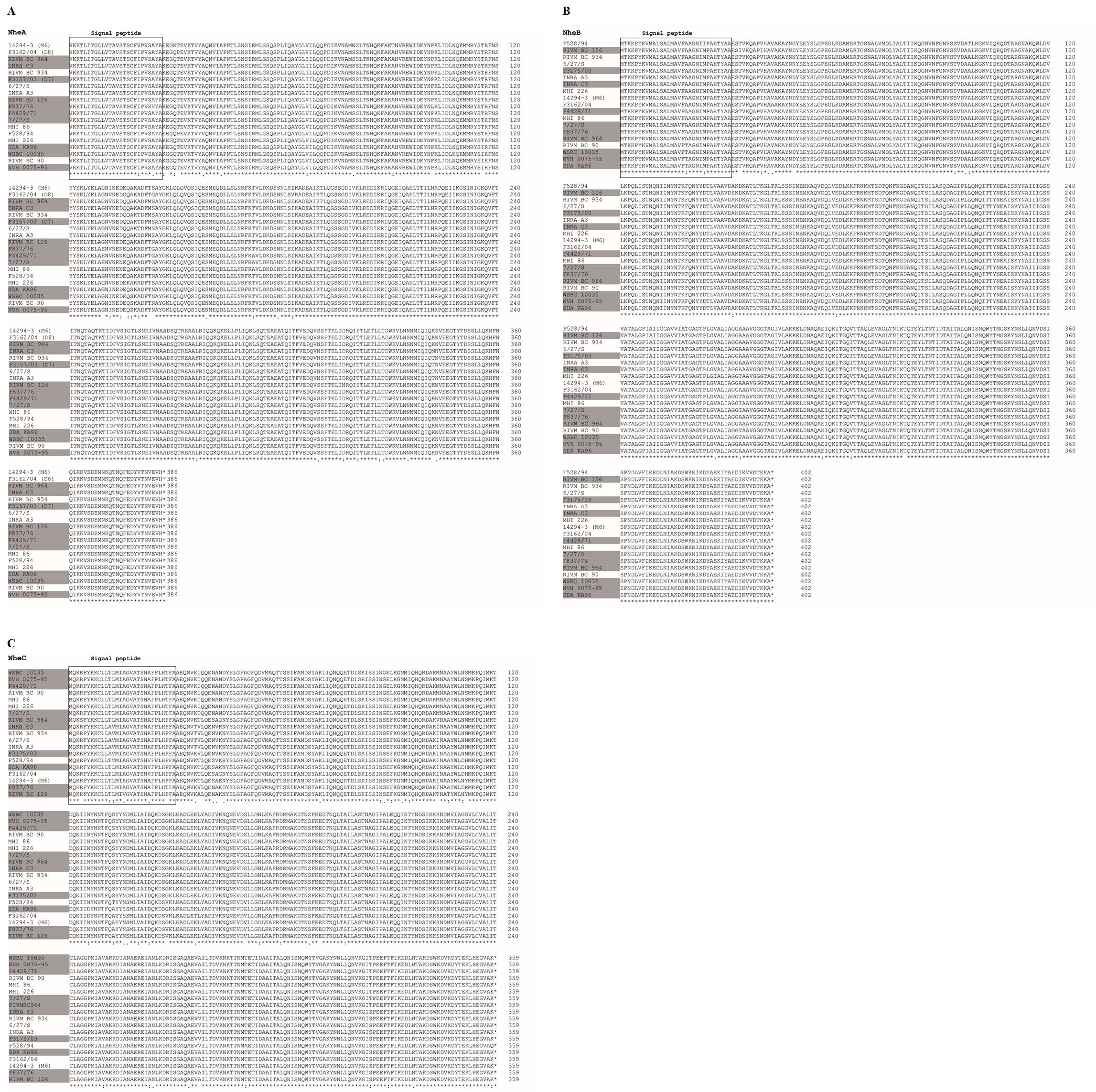

Supplement: Supplementary file 5 [file Image2.JPEG]

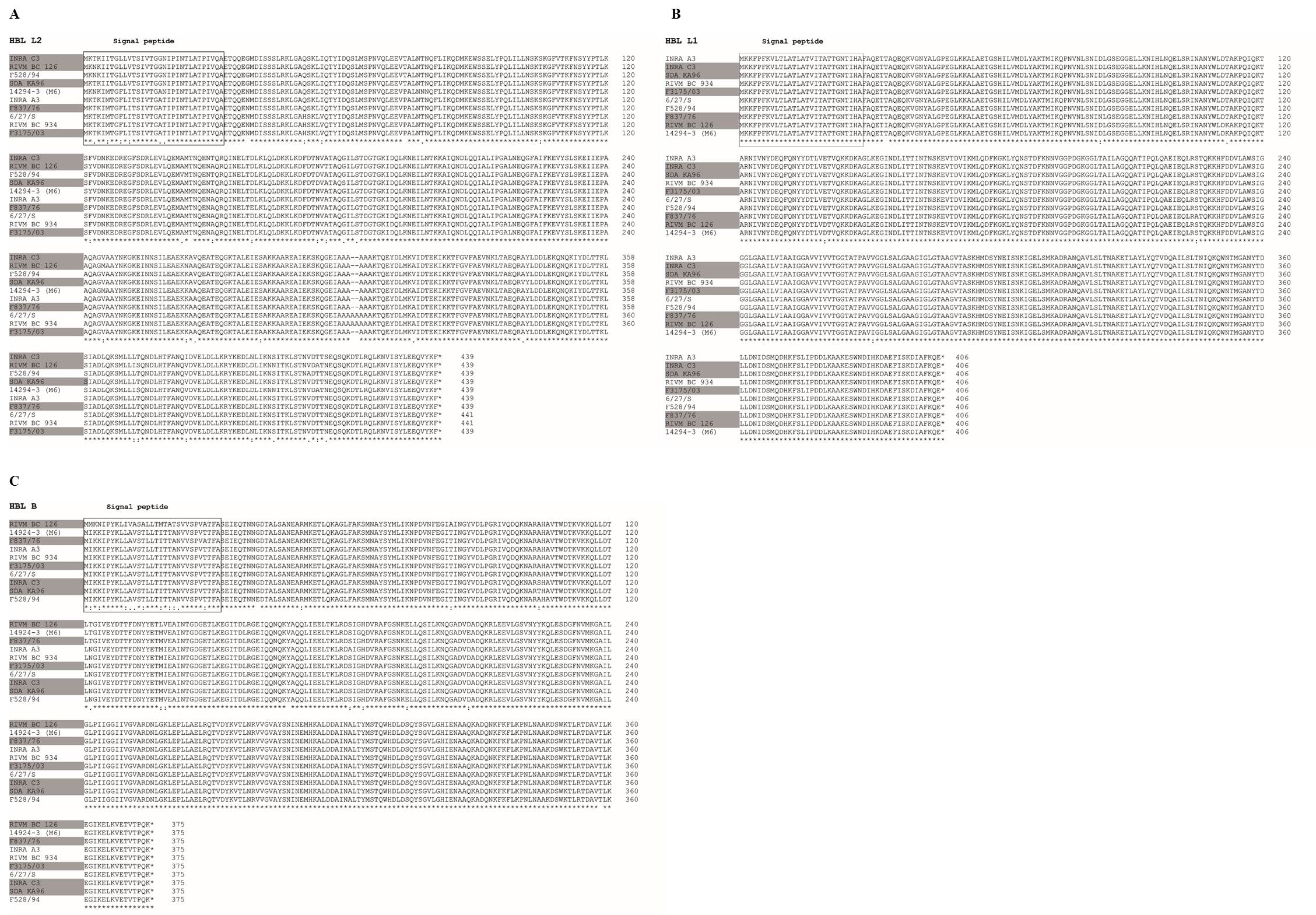

Supplement: Supplementary file 6 [file Image3.JPEG]

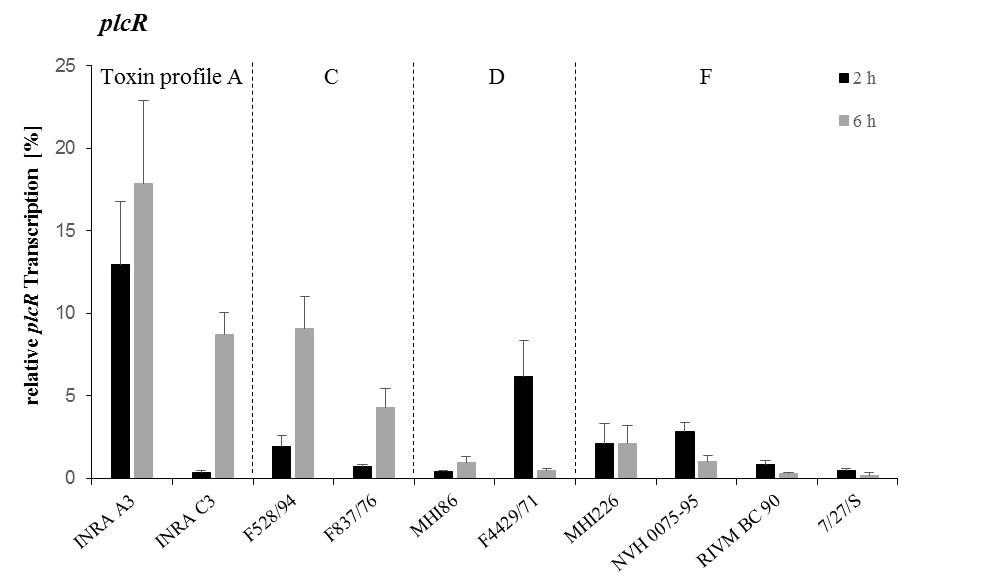

Supplement: Supplementary file 7 [file Image4.JPEG]
